# Polarimetry as Applied to Urinalysis

**Published:** 1911-11

**Authors:** H. F. Lichtenberg


### Polarimetry as Applied to Urinalysis

By H. F. LICHTENBERG, A.C.

THE principle of polarimetry or optical saccharimetry depends upon the fact that certain substances possess the power of rotating a beam of polarized light either to the right or to the left of the normal. That is to say, that if light, vibrating in a certain plane, is passed through a solution of an optically active substance it is thereby caused to vibrate in another plane which is at an angle to the first plane. This angle of rotation is directly proportional to the amount of active substance in solution.

The polariscope which is used most generally at present is known as the Soleil Ventske and is graduated to read the percentage of sucrose directly. Therefore, the following methods were worked up for use with this instrument. The older form of this instrument was graduated to read in angular degrees and so for the benefit of those who still possess this type of polariscope, a formula is given so that the said methods can also be used in connection with the older instrument. Lately a new form of "Polarizing Saccharimeter" has been designed by Ultzmann. It is intended especially for Urinary work. Its principal difference from the other instrument is, that it reads dextrose directly and uses solar light for illumination. Thus the field must necessarily be a colored one instead of a monochromatic one. When the tube is empty the field is of even color, but when it contains a dextrose solution, two colors, which are separated by a sharp line, are visible. In order to read the rotation it is necessary to turn the adjustment screw until the "transition tint" is obtained.

The greatest objection to the instrument is that this point is more difficult to determine than an even illumination as in the half shade instrument, because the suceptibility to delicate tints varies with the eyes of the operator and thus an extra error is introduced. But anybody can tell the difference between light and darkness or when the field is evenly illuminated. This is only one of many[2] reasons why the half shade instrument is to be preferred and therefore the author has worked up his methods to apply principally to the latter instrument.

All of the standard text books on Urinalysis or Clinical Diagnosis give a little space to polarimetry but in perusing them we soon find that they, one and all, attempt to describe some form of the instrument in detail, as to theory, construction, etc. Now while this is all well and good it is only a waste of space; first, because better descriptions can be found in standard text books on physics; second, because the methods for use as given in connection with the description of the instrument are just the converse. They are so brief and so indefinite that they can be applied to only a very few samples of urine, i.e., to those only which are very light in color and contain no albumin. Some of the books give various methods of clarifying and decolorizing the urine intended for polarimetry. These methods are very unreliable and uncertain and also applicable only to some urines. For example, several books direct to decolorize with animal charcoal, which, as shown by Scheibler,[1] absorbs sugar in proportion to the amount of charcoal used and thus the result of polarization will be too low. Others make statements which are, to say the least, absurd, i.e., Emmerson[2] says that Lead sub-acetate, when used as a decolorizing agent, precipitates some dextrose. In order to prove the fallacy of this statement I examined five samples of urine,—each a normal urine made up with a known weight of dextrose—by the gravimetric method both before and after decolorization, and found absolutely the same percentage of sugar before and after decolorizing.

Therefore, I judge that Emmerson probably forgot to make a correction for the amount of lead subacetate solution added, which mistake would necessarily give low results.

Believing that polarimetry could be applied to urinalysis in a simple practical way, the author has made an exhaustive research (both literary and experimental) on the subject and has found methods whereby, not only sugar but also albumin may be determined by the polariscope. These methods have been tried on quite a number of samples and can be recommended as being perfectly reliable in every way when the directions as given in the following pages are carefully followed.

### DETERMINATION OF DEXTROSE.

The sugar which is found in urine in diabetes is the dextrogyrate modification of glucose, namely dextrose and may be determined as follows:

The general proceedure is to fill the two decimeter observation tube with the filtered urine. The tube is placed in the instrument (illuminated by a yellow or sodium light), with the scale set at zero, and if the urine contains sugar, the field will be unevenly illuminated. That is, a sharp line will be seen running vertically through the field, dividing it into two equal halves, one of which is brightly illuminated, while the other is dark. The adjustment screw is now turned until both halves of the field are evenly illuminated (or in the case of Ultzmann's instrument, until the two halves are of even color.) The scale is now read and noted. Then the scale should be turned back to zero and another reading made. This should be repeated until three readings have been made. The arithmetical mean of these is then taken and the calculation made as follows:

Since the Soleil Ventske instrument is graduated to give the percentage of sucrose directly, and since the specific rotation of sucrose is more than that of dextrose, it is obvious that a correction must be made.

Specific rotation is usually designated by [$a$] and D indicates that the D, ray or sodium (yellow) light is used.

This is important because specific rotation varies with the wave length of light. In other words a monochromatic light is absolutely essential and the sodium light which corresponds to the D, ray gives best results.

[$a$]d=66.48 for Sucrose and [$a$]d=52.3 for Dextrose.

Thus it can be seen that it is only necessary to multiply the reading noted on the sucrose scale, by the constant (.7867) to obtain the reading in terms of dextrose or the number of grams of dextrose per hundred c.c. of urine. For all practical purposes, however, it is perfectly correct to call gms. per 100 c.c. percentage because the difference in specific gravity between distilled water and urine is not enough to introduce an error large enough to be detected by the polariscope which is not sensitive to quantities of sugar smaller than .2 per cent.

The following experiment will serve to illustrate this:

Three grams of pure dextrose dissolved in normal urine and made up to 100 c.c. with the same urine gave these readings, viz., 3.9, 3.8, 3.7 in terms of sucrose, then by calculation of the mean we have 3.8x.7867=2.989 per cent. *in terms of dextrose.*

Now to make corrections for the specific gravity which was 1,020 the result is as follows. 100 c.c. of the solution would weigh 102 grams. Then 3 grams: 102 gms. as x :100, 102x=300, x=2.94 gms. or per cent. of dextrose in solution (corrected for gravity). Therefore, it can be seen that the error (between

2.98 per cent. and 2.94 per cent.) is too slight to be detected by the polariscope and thus it is not necessary to correct for gravity.

The directions given thus far are not sufficient to apply to all urines. For example, a highly colored or dark urine absorbs so much light as to make the field very dim and indistinct and thus the readings either cannot be made at all, or they will be inaccurate and therefore of no value.

There are two remedies. First, an observation tube of shorter length (one decimeter for example) may be used and then the result multiplied by 2 or if the field is still too dim, the second remedy may be used. Second, the urine may be clarified and decolorized as follows. With a graduated pipette, put into a 100 c.c. volumetric flask exactly 10 c.c. of Official Lead Sub-acetate solution and then add urine until the total volume is exactly 100 c.c. Agitate this gently and allow to stand for about five minutes. (The Lead Sub-acetate precipitates phosphates, chlorides, sulplates, etc. and carries down with it most or all of the coloring matter.) The solution should now be gently shaken and then filtered through a dry filter. The first few drops of the filtrate are apt to come through cloudy. If this is the case, the filtrate must always be poured back through the same filter until it is clear. Two filterings (always using the same filter) are usually sufficient. The two decimeter observation tube is now filled with the clarified urine (which should be almost colorless) and read as before. Then the final result will be the number of grams in 90 c.c. of urine and in order to obtain the per cent. of grams in 100 c.c., it is necessary to multiply this reult by 1.10 or increase the reading by 1-9.

If it should be found necessary, both of these remedies may be combined into one. That is, first decolorize as directed, then fill a one decimeter observation tube, read and calculate to dextrose. This will be grams per 90 c.c. Then calculate to grams per 100 c.c. and finally multiply by 2 as the two decimeter tube is standard. This combination makes it possible to determine the sugar in any urine if present in quantity larger than 0.2 per cent. which is sufficient for most practical or clinical purposes.

DETERMINATION OF ALBUMEN.

Albumin exercises a left rotating power over polarized light and thereupon depends the principle of its determination by the polariscope.

The general proceedure is the same as for dextrose up to the calculation, which is as follows. The specific rotation of serum albumin is, $[a]d = -53.$ and

$$\frac{[a]_D = -53.}{[a]_D = 66.48} = 0.7972$$

constant for changing the albumin reading on the sucrose scale to terms of albumin. The calculation is the same as for converting dextrose.

This theoretical constant was proved to be correct in the following way.

Experiment. Five samples of albuminous urine were examined by the gravimetric method and with the polariscope. Then dividing the quantity of albumin found by the percentage read on the sucrose scale, as per formula, a constant was obtained which agreed with the theoretical constant. Then conversely, the percentage by albumin read on the sucrose scale was multiplied by the constant and results which corresponded very closely with those obtained by the gravimetric method were obtained.

COMPARISON BETWEEN THE GRAVIMETRIC METHOD AND POLARISCOPIC METHOD, FOR ALBUMIN.

| Percentage of albumin found by the gravimetric method | Percentage of albumin in terms of Sucrose | Constant or factor found by calculation | Percentage of albumin corrected with factor |
|---|---|---|---|
| 1.663 | 2.0 | 0.7972 | 1.5944 |
| 1.400 | 1.8 | .7972 | 1.41496 |
| 1.801 | 2.3 | .7972 | 1.833356 |
| 2.190 | 2.7 | .7972 | 2.15244 |
| 2.661 | 3.2 | .7972 | 2.55104 |

By comparing columns one and four it can be seen that the results obtained by both methods are practically the same and thus the polariscope furnishes a simple and accurate means for determining albumin.

When it is necessary to clarify the urine for determining the albumin by the polariscope, the Lead Sub-acetate solution aforementioned cannot be used because some albumin is precipitated by the lead sub-acetate solution and thus the results would be low and inaccurate.

Therefore, I have used the solution known as Alumia Cream which does not precipitate the albumin. It is used in the same way as the sub-acetate solution but with high colored urines it is usually necessary to pass the filtrate through the same filter several times. A 50 m.m. observation tube can often be used to advantage. Then the result must, of course, be multiplied by four.

Methods for removing albumin from the urine:

The removal of albumin by heat: If the urine contains more than a very slight trace of albumin it should always be removed before testing for sugar since the albumin either enters into combination with the reagents or reacts with them in such a manner as to render the tests unreliable. The best method for the removal of albumin is to coagulate it by heat; this should be applied in connection with both qualitative and quantitative analysis.

1. For Qualitative Tests. Take one-third of a test tube of urine, add one drop of dilute acetic acid and about one-half gram of sodium chloride. Boil the whole mixture thoroughly. If a flocculent precipitate does not form, add at intervals drop by drop more acetic acid, heating the mixture after each addition until a distinct flocculent coagulum forms. Filter; the filtrate should be perfectly clear and practically free from albumin.

2. For Quantitative analysis: Put into a beaker 50 c.c. of urine, add a little salt, as before, and two or three drops of dilute acetic acid and boil thoroughly. If a flocculent coagulum of albumin does not appear, add more acid, drop by drop, with constant stirring and continuing the heat until such a coagulum forms.

Filter; the filtrate should be free from precipitate; and wash once or twice with water. Allow the filtrate and wash water to run into a 50 c.c. volumetric measuring flask, and then add sufficient water to make the original volume (50 c.c.). Mix the contents of the flask thoroughly and use for quantitative tests.

In removing albumin by heat a flocculent coagulum should be obtained in all cases, and this is accomplished when the urine has a faintly acid reaction (preferably with acetic acid). In case a flocculent coagulum is not obtained, the filtrate will be more or less turbid, the turbidity being due to the finely divided precipitate of albumin. Such a turbid filtrate is unfit for further tests.

2. Solution for clarifying and decolorizing urines to be polarized for sugar. The Official Lead Sub-acetic solution is used and is prepared as follows: Prepare by boiling 430 grams of normal lead acetate, 130 grams of litharge, and 1,000 c.c. of water for half an hour. Allow the mixture to cool and settle and dilute the supernatant liquid to 125 specific gravity with recently boiled water. Solid lead sub-acetate may be substituted for the normal salt and litharge in preparation of the solution.

3. Solution for clarifying and decolorizing urines to be polarized for albumin. Preparation of the Official Alumina Cream. Prepare a cold saturated solution of alum in water and divide into two unequal portions. Add a slight excess of ammonium Hydroxide to the larger portion and then add by degrees the remaining alum solution until a faintly acid reaction is secured.

Formula for calculating the percentage of dextrose from readings made on polariscopes graduated in angular degrees.

$$D=\frac{a \times 100}{[a]_D \times l}$$

D=the amount of dextrose in 100 c.c. of the solution.
a=the angle of rotation.
l=length of tube used in making observations.
$[a]_D^{20}=+53°$ equals the specific rotation of dextrose at 20°C.

If an observation tube of 189.4 m.m. is used with the above mentioned instrument, each angular degree of rotation will correspond to 1 per cent. of dextrose. Thus the above formula need not be taken into account.

REFERENCES:

U. S. Department Agriculture, Bureau of Chemistry, Bulletin 107, 1908.

Landolt, Optische Drehungsvermogen, Braunschweig, 1898.

Scheibler: Idem 1870 p. 218.

Clinical Diagnosis p. 181 (Emmerson).

